# Evolving Devices and Material in Transcatheter Aortic Valve Replacement: What to Use and for Whom

**DOI:** 10.3390/jcm11154445

**Published:** 2022-07-30

**Authors:** Mauro Chiarito, Alessandro Spirito, Johny Nicolas, Alexandra Selberg, Giulio Stefanini, Antonio Colombo, Bernhard Reimers, Annapoorna Kini, Samin K. Sharma, George D. Dangas, Roxana Mehran

**Affiliations:** 1 The Zena and Michael A. Wiener Cardiovascular Institute, Icahn School of Medicine at Mount Sinai, One Gustave L. Levy Place, New York, NY 10029, USA; mauro.chiarito@humanitas.it (M.C.); johny.nicolas@mountsinai.org (J.N.); alessandro.spirito@mountsinai.org (A.S.); alexandra.selberg@mountsinai.org (A.S.); annapoorna.kini@mountsinai.org (A.K.); samin.sharma@mountsinai.org (S.K.S.); george.dangas@mountsinai.org (G.D.D.); 2 Department of Biomedical Sciences, Humanitas University, Via Rita Levi Montalcini 4, 20090 Pieve Emanuele, Italy; giulio.stefanini@hunimed.eu (G.S.); colombo@emocolumbus.it (A.C.); bernhard.reimers@humanitas.it (B.R.); 3 Istituti di Ricovero e Cura a Carattere Scientifico, Humanitas Research Hospital, Via Alessandro Manzoni, 56, 20089 Rozzano, Italy

**Keywords:** transcatheter aortic valve replacement, aortic stenosis, aortic regurgitation, self-expanding valve, balloon-expandable valve, transcatheter heart valve

## Abstract

Transcatheter aortic valve replacement (TAVR) has revolutionized the treatment of aortic stenosis, providing a viable alternative to surgical aortic valve replacement (SAVR) for patients deemed to be at prohibitive surgical risk, but also for selected patients at intermediate or low surgical risk. Nonetheless, there still exist uncertainties regarding the optimal management of patients undergoing TAVR. The selection of the optimal bioprosthetic valve for each patient represents one of the most challenging dilemmas for clinicians, given the large number of currently available devices. Limited follow-up data from landmark clinical trials comparing TAVR with SAVR, coupled with the typically elderly and frail population of patients undergoing TAVR, has led to inconclusive data on valve durability. Recommendations about the use of one device over another in given each patient’s clinical and procedural characteristics are largely based on expert consensus. This review aims to evaluate the available evidence on the performance of different devices in the presence of specific clinical and anatomic features, with a focus on patient, procedural, and device features that have demonstrated a relevant impact on the risk of poor hemodynamic valve performance and adverse clinical events.

## 1. Introduction

Aortic stenosis (AS) is the most common acquired heart valve disease in developed countries, affecting up to 10% of elderly patients [1]. The prevalence of AS is expected to increase over the next decades with the increasing life expectancy in most developed countries. Indeed, the global number of people older than 80 is foreseen to triple and surpass 400 million by 2050, with AS prevalence expected to be growing at a similar rate [2]. AS has a 50% mortality rate at 5 years from symptom onset if left untreated [3]. Until recently, surgical aortic valve replacement (SAVR) represented the only definitive treatment for patients with AS, as medical therapy can only mitigate symptoms. Nonetheless, considering the frailty and the relevant burden of comorbidities of many elderly patients with symptomatic AS, a considerable portion of this population was left untreated, due to high or prohibitive surgical risk. Transcatheter aortic valve replacement (TAVR) was originally conceptualized in the early 1990s [4], largely inspired by the pioneering experiences in the field of percutaneous transluminal coronary angioplasty. Following different studies in animal models [5], the first TAVR procedure was performed in 2002 for the treatment of AS in a patient with several comorbidities and cardiogenic shock. [6] Since then, TAVR has revolutionized the treatment of patients with severe, symptomatic AS, as randomized controlled trials have shown similar, if not superior, outcomes following TAVR as compared with SAVR in selected patients [7,8]. TAVR first emerged as a plausible treatment option for patients with AS at high or prohibitive surgical risk. Due to major advances in TAVR technologies, subsequent trials have shown that it is a safe and effective alternative to surgery for patients at intermediate-to-low surgical risk [8,9]. The number of TAVR procedures is rapidly increasing, and the continuous expansion of the population deemed suitable for TAVR [10,11] has corresponded with an impressive constant evolution in TAVR devices and materials. Indeed, these advances have significantly reduced periprocedural complications, making it safe to shorten hospital stay and improve long-term outcomes [12,13]. Moreover, these progresses have resulted in a wide armamentarium at our disposal, including bioprostheses presenting different dimensions, designs, and deliverability, providing the opportunity to select a device based on each patient’s clinical and anatomic characteristics. In addition, as TAVR comes of age, clinical indications for TAVR are gradually expanding, from elderly and comorbid patients affected by calcific AS to younger patients, with bicuspid aortic valve, bioprosthesis degeneration, and/or aortic regurgitation (AR), all conditions that potentially require devices with specific features. The present review aims to describe currently available TAVR devices and to provide an overview of the design features that should guide the selection of the most appropriate device for each patient, presenting the pros and cons of each device and how their features might mitigate the risk of specific adverse events.

## 2. Types of TAVR Devices

Since the first transcatheter implantation of the aortic Cribier–Edwards valve (Edwards Lifesciences, Irvine, CA, USA) in 2002 [6], several new transcatheter heart valves (THV) were introduced and approved for clinical use. The development of a safe and efficacious THV is technically challenging, as the valve must be crimped before implantation and then deployed over a heavily calcified aortic valve. Over time, improvements in valve design, materials, and delivery systems facilitated the implantation of the valve in the desired position, and decreased procedural and periprocedural complications [14]. For example, the sheath size for the delivery system was reduced from 24 to 12–14 Fr to enable implantation through a narrower vascular access and to reduce vascular complications. Sealing technologies, such as an outer skirt or a pericardial wrap, contributed to reduce the rates of paravalvular leak (PVL). Moreover, frame height was decreased, and the sizes of frames’ upper cells were enlarged to facilitate continued coronary access [15,16].

**Figure 1:**
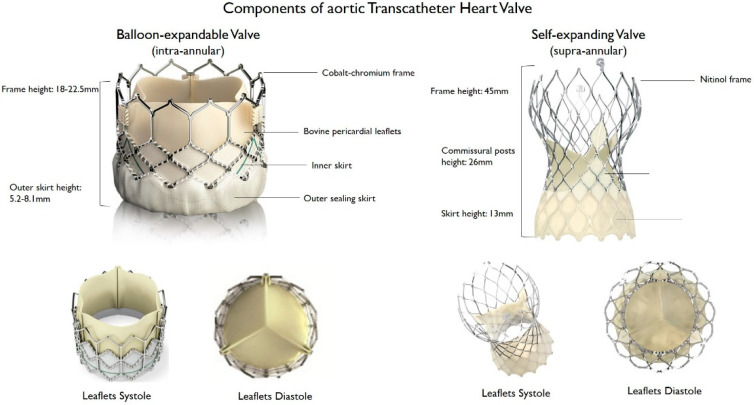
Main features of balloon–expandable and self–expanding aortic transcatheter heart valves.

THV consist of a three-leaflet valve, made of bovine or porcine pericardium or polymeric material, mounted on a radiopaque metallic scaffold (frame), made of stainless steel, nitinol, or cobalt–chromium, and wrapped by an outer sheath (skirt or wrap)—in pericardial or polymeric material—to increase the surface area contact between the device and the native valve, and mitigate the risk of significant PVL (Figure 1). According to the position of the prosthetic leaflets relative to the native valve annulus, the THV is labeled as supra- or intra-annular. Supra-annular valves usually result in a larger effective orifice area (EOA) and lower transvalvular aortic mean gradients than intra-annular THV, which have a lower frame height that eases coronary access. Some THVs can be recaptured and repositioned after implantation, while other THV are non-repositionable after deployment. The delivery systems differ regarding the degree of flexion of the distal catheter and sheath diameter. The most common delivery approach is transfemoral, but other access routes (i.e., trans-subclavian, transaortic, transapical, transcarotid and transcaval) are used, as iliofemoral and aortic vessel diseases are commonly present in TAVR patients.

**Figure 2:**
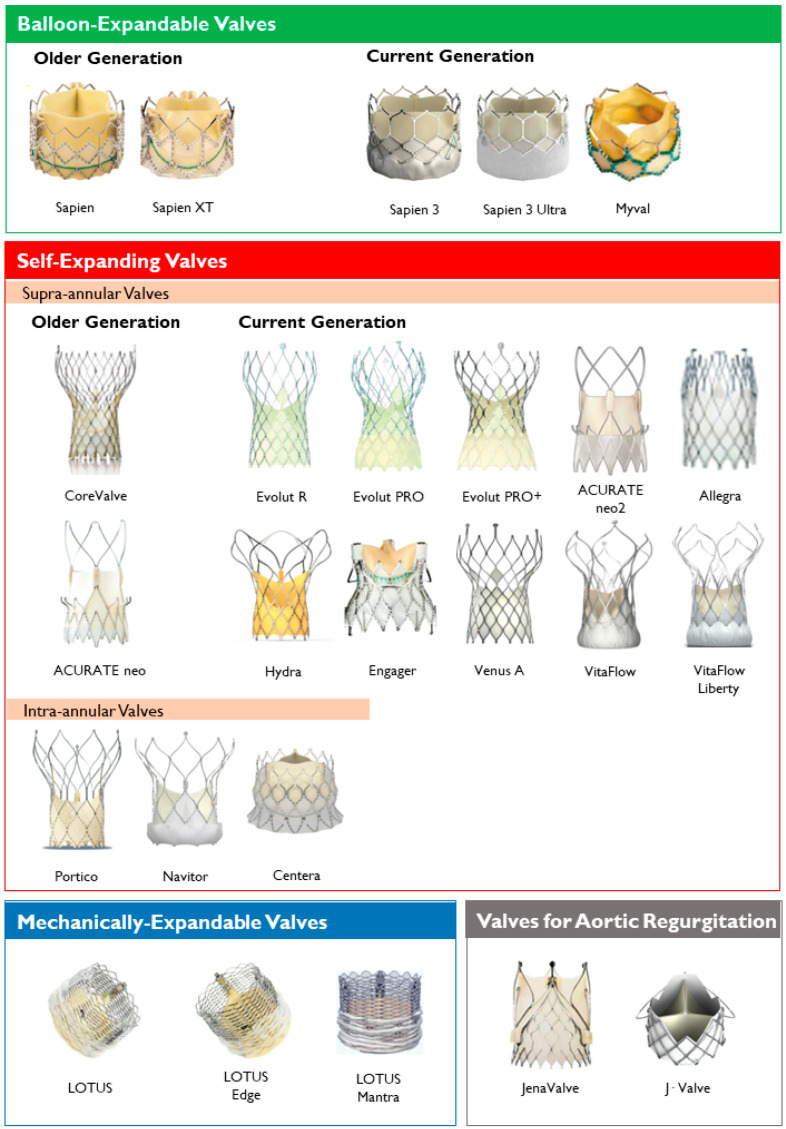
Transcatheter heart valves, stratified by mechanism of the valve frame expansion and leaflets position.

Lastly, the most common classification of THVs is based on the mechanism of the valve frame expansion (Figure 2) as self-expandable (SE), balloon-expandable (BE) or mechanically expandable. Detailed information on each THV are provided in Table 1.

### 2.1. Balloon-Expandable Valves

The first implanted THV (Cribier-Edwards) [6], SAPIEN devices (Edwards Lifesciences), and the Myval THV (Meril Life Sciences, Gujarat, India) belong to the BE group.

The expansion of these valves requires balloon inflation during rapid ventricular pacing, which may not be well tolerated by patients with reduced left ventricular ejection fraction (LVEF) or impaired renal function. All BE-THV are intra-annular, are not repositionable and have a lower stent frame profile, facilitating coronary access as compared with SE-THV. Furthermore, the delivery system allows for greater steerability than SE- or ME-THV, helping in valve implantation in patients with challenging vascular anatomy, such as in case of horizontal aorta, defined as an aortic angulation >60°.

The SAPIEN 3 consists of a trileaflet bovine pericardial valve mounted in a cobalt-chromium frame with an outer seal cuff to reduce PVL; in the SAPIEN 3 Ultra, frame height was increased to further reduce the rate of PVL [17]. The SAPIEN 3 and SAPIEN 3 Ultra are FDA approved [18].

The Myval THV (Meril Life Sciences, Gujarat, India) obtained the CE mark in 2019 but is not yet FDA-approved; it consists of a nickel–cobalt alloy (MP35N) frame, a trileaflet valve of bovine pericardium tissue and an external polymeric sealing cuff [19,20].

### 2.2. Self-Expanding Valves

The group of SE-THV includes a wider range of devices from different companies. The majority of SE-THV are supra-annular, resulting in a higher EOA, lower gradients, and lower rate of severe prosthesis–patient mismatch (PPM). Rapid ventricular pacing during implantation is not mandatory and most SE-THV are repositionable and/or retrievable, at the expense of limited steerability. Of note, the greater frame height of SE-THV may make coronary access more challenging.

The Medtronic Corevalve (Medtronic, Sunnyvale, CA, USA) was the first self-expanding THV developed, and together with its subsequent generations—Evolut R, Evolut PRO and Evolut PRO+—represents the most studied and commonly implanted SE-THV and has CE and FDA approval. The Evolut PRO+ added an outer porcine pericardial tissue wrap that increases surface area contact and tissue interaction between the THV and the native aortic annulus. Of note, the frame of Medtronic SE-THV, due to its higher radial force, exerts a higher pressure on the membranous septum and the conduction system than BE-THV, resulting in considerable risk of conduction abnormalities requiring PPI [21].

The ACURATE neo (older generation) [22] and ACURATE neo2 (newer generation) [23] produced by Boston Scientific, have similar characteristics to the Evolut THV, except for the fact that implant depth can be controlled due to its top-down deployment, it is non-repositionable, and that predilation of the aortic valve is strongly recommended.

Other SE-THV with CE but not FDA approval include: Allegra (NVT AG), whose grip uses a “squeeze-to-release” mechanism, avoiding any rotation during the entire implantation, performed in a stepwise manner [24,25]; Hydra (Vascular Innovations Co., Ltd., Nonthaburi, Thailand) which includes a mechanism for recapturing during release [14]; Engager (Medtronic), and Venus-A valve (Venus Medtech, Hangzhou, China) [26]. VitaFlow (Microport, Shanghai, China) and its subsequent iteration VitaFlow Liberty™ are novel THVs manufactured in China, for which the CE approval is ongoing [27,28].

Among SE-THV, some are intra-annular, such as Centera (Edwards) [29], Portico (Abbott Structural Heart, Westfield, IN, USA) and its iteration Navitor (Abbott Structural Heart). The Portico valve (Abbott) is a self-expanding, fully resheathable and retrievable valve with leaflet geometry designed to function in both round and elliptical configurations. The annular positioning facilitates the engagement of coronary ostia after implantation [15,30]. This valve is CE- and FDA- (September 2021) approved. The Navitor THV has as a key innovation in an active outer fabric cuff designed to reduce the PVL.

SE-THV with supra-annular designs are particularly indicated in patients with small or severely calcific annulus, TAVR-in-SAVR, and both supra- and intra-annular valves are indicated in patients at risk for poor tolerance to rapid pacing.

### 2.3. Mechanically-Expandable Valves

The expansion of mechanically expandable-THV is mediated by a mechanical controlled system and usually does not require rapid ventricular pacing. These valves are intra-annular, fully repositionable, and retrievable. This group included LOTUS (older generation), LOTUS Edge and LOTUS Mantra (newer generation, all produced by Boston Scientific, but currently recalled due to issues with the product delivery system) [31].

### 2.4. Valves with Active Fixation Mechanisms

In recent years, valves equipped with an active fixation mechanism were developed. The anchor mechanism enables fixation of the prosthesis onto the native valve leaflets, providing stability in the context of non-calcified native valves and allowing implantation in patients with aortic regurgitation.

The JenaValve (JenaValve Technology, München, Germany), a porcine pericardial valve in a low-profile nitinol frame with a paperclip-like fixation mechanism, is currently the only THV with a CE mark for use in patients with aortic regurgitation [32]. The J-Valve (JC Medical) is another device that can be employed for the treatment of native AR, as well as AS, and is currently being evaluated in an early feasibility study in the US [33]. Two further valves from China, the Venus-AVR valve (Venus Medtech) and VitaFlowVR (Microport), are at an advanced stage of development with high rates of procedural success in the challenging cohort of patients with a bicuspid aortic valve [15,26].

## 3. Adverse Events after TAVR

Despite significant advances in TAVR technologies, there are still opportunities for improvement in the prevention of periprocedural and mid- to long-term adverse events. The rates of most of these events have dropped significantly over the past few years (Table 2), but their prognostic impact warrants caution and meticulous procedural planning to optimize post-TAVR outcomes.

### 3.1. Stroke and Subclinical Leaflet Thrombosis

Stroke, the risk of which is highest in the periprocedural period and within the first 3 months after TAVR, remains a feared complication associated with up to 10-fold increase in mortality rates within 2 years of the index procedure. The incidence of stroke during and after TAVR across large randomized clinical trials ranges from 0.6% to 8%, depending on the surgical risk of study participants, type of bioprosthetic valve implanted, and the era during which the study was conducted [34]. Indeed, early studies comparing TAVR versus SAVR in high-risk patients with AS revealed significantly higher rates of stroke with TAVR than SAVR. In the randomized Placement of Aortic Transcatheter Valves (PARTNER 1) trial, the rates of stroke/transient ischemic attack reached 5.5% at 30 days and 7.7% at two years after TAVR, compared with 2.4% at 30 days and 4.9% at two years after SAVR [35]. However, subsequent studies published over the past few years showed similar or even lower rates of stroke following TAVR (0.3% and 2.1% at 30 days in PARTNER 3 and Evolut Low Risk trials, respectively) as compared with SAVR (2.4% and 1.9% at 30 days in PARTNER 3 and Evolut Low Risk trials, respectively) [8,9]. Similarly, real-world studies reported 1-year stroke rates of 2% to 3%, which have remained stable over the last two decades [36,37]. This is largely due to significant advances in bioprosthetic valve design and device delivery technologies, increased operator experience, and expansion of TAVR indications to intermediate- and low-risk patients. The risk of stroke seems to be highest in the periprocedural (within 48 h after the procedure) and 30-day period after TAVR, with a steady decrease over time [38]. It is estimated that up to 84% of patients undergoing TAVR have new embolic cerebral insults detected by magnetic resonance imaging [39]. Although most of these insults does not result in acute neurologic deficit and are no longer detected by magnetic resonance within months, the occurrence of silent cerebral ischemic lesions has been shown to be associated with a more pronounced transient neurocognitive decline early after TAVR and with lower recovery at follow-up [40]. In this setting, cerebral protection devices represent a possible solution to reduce TAVR-related stroke. However, data supporting its use are limited as most TAVR randomized studies were underpowered for uncommon clinical end points such as stroke [41]. The risk of stroke and embolic events remains elevated for the following 3 months and matches the risk of age-matched patients after this timeframe [42]. If acute events are mainly related to the procedure itself, subacute and late thromboembolic events are usually due to activation of a coagulation cascade linked to pre-existing or new-onset atrial fibrillation and dislocation of clots from the THV [38].

With the expansion of TAVR indications to a broader and younger patient population, bioprosthetic valve durability and optimal function have become a central consideration in contemporary management of severe symptomatic AS. Observational data suggest a high rate (up to 30%) of subclinical leaflet thrombosis with or without motion abnormalities in patients undergoing aortic valve replacement [43,44]. Isolated subclinical leaflet thrombosis as detected on multidetector computed tomography is known as hypoattenuated leaflet thickening. A more serious form of valvular dysfunction occurs when hypoattenuated leaflet thickening is associated with hypoattenuation affecting motion. Both phenomena have been detected on imaging in patients receiving various types of TAVR valves and surgical bioprostheses [45,46]. Unfortunately, the clinical significance of these complications with regard to progression to clinical thrombosis, stroke or transient ischemic attack, and valve durability is still debated and continues to be a matter of ongoing research.

### 3.2. Prosthesis-Patients Mismatch

As PPM is defined by an effective prosthesis area smaller than a native human valve [47], some degree of PPM is unavoidably present in all patients undergoing aortic valve replacement. Of note, prognostic implications of PPM, such as valve dysfunction and structural deterioration, persistence of left ventricular hypertrophy, heart failure-rehospitalization, and mortality, may develop only if the mismatch reaches a critical threshold [48,49].

According to the Valve Academic Research Consortium 2 criteria, PPM is defined as moderate with an indexed EOA between 0.85 and 0.66 cm^2^/m^2^ of body surface area, and severe with an of EOA < 0.65 cm^2^/m^2^ [50]. Post-hoc analysis of the PARTNER and U.S. CoreValve High Risk Study has shown a reduced rate of PPM with TAVR as compared with SAVR (e.g., 6.2% vs. 25.7% in the U.S. CoreValve High Risk Study) [51,52], irrespective of the implantation of stentless or stented surgical valves [53], a difference related to thinner struts and lack of sewing ring on TAVR bioprostheses. This difference was maintained in the setting of intermediate surgical risk population and in the Evolut Low Risk Trial. Of note, no differences were reported in terms of severe PPM in the PARTNER 3 trial (4.6% vs. 6.3%, TAVR vs. SAVR, respectively) [54], as larger valves were implanted in the surgical arm, as more aggressive root enlargement resulted in improved hemodynamics in the surgical group of the PARTNER 3 trial as compared with the previous PARTNER studies [9].

### 3.3. Paravalvular Leak

PVL is generally a result of an incomplete seal between the bioprosthetic valve and aortic annulus during valve implantation and is largely dependent on specific valve design and anatomic features. The rates of PVL are substantially higher after TAVR than SAVR, owing to the technical aspects of each procedure [55]. Although the majority of post-TAVR PVL is mild, moderate or severe PVL do occur frequently and are strongly associated with mortality [56]. Importantly, even mild PVL leads to intense sheer stress and alters the conformation of high-molecular-weight multimers of von Willebrand factor by unfolding the high-molecular-weight multimers and exposing them to proteolytic cleavage. The destruction of Willebrand multimers impairs the hemostatic role of von Willebrand factor, ultimately increasing the bleeding risk and subsequently mortality rates in patients with PVL [57].

As a result, the evolution of bioprosthetic valve design over the past few years has been primarily focused on PVL reduction. These improvements led to a progressive reduction in the rates of moderate and severe PVL, as seen in recent low-risk TAVR trials (0.8% and 3.5% at 30 days in PARTNER 3 and Evolut Low Risk trials, respectively) [8,9]. Furthermore, the latest generation BE SAPIEN 3 Ultra seems to reduce not only moderate and severe PVL, but also mild PVL, as compared with its predecessor the SAPIEN 3 [58]. Similar outcomes have been reported for the newer generation SE Evolut PRO+ versus the Evolut R THV at 30 days after TAVR [59]. Overall, the newer generation self-expanding valves seem to be associated with lower PVL rates than balloon-expandable valves (1.5% versus 3.4% at 30-day follow-up) [60].

### 3.4. Complex Post-TAVR Coronary Access

The need for post-TAVR coronary angiography and revascularization is expected to increase with the growing population of younger TAVR patients. Due to native leaflets that remain in place and difficulty ensuring commissural alignment with existing valves and high THV frame, TAVR is frequently associated with a considerable risk of delayed coronary occlusion (0.2% in overall population, but up to 3.5% in patients at high risk, such as those undergoing valve-in-valve (ViV) procedures) [61,62] and with challenging coronary re-access [63]. In contrast, resection of native valve leaflets during SAVR along with an optimal commissural alignment of the bioprosthetic valve allow for an easier access to coronary ostia following valve replacement. The degree of challenge in coronary access correlates well with the design of the implanted THV. For example, coronary access after balloon-expandable valve implantation is relatively easier than after self-expanding valve implantation due to the shorter stent frame and sub-coronary position of the former [64].

Of note, the bioprosthetic or native aortic scallop intentional laceration to prevent iatrogenic coronary artery obstruction during TAVR (BASILICA) has emerged as a safe and effective option to limit the risk of coronary obstruction and facilitate the access to coronary ostia, especially in patients undergoing ViV.

### 3.5. Vascular Complications

TAVR is preferably performed using the femoral access route [10]. Early and late vascular complications are not infrequent after TAVR and can occur in up to 10% of patients [65]. Vascular complications include aortic dissection or rupture, vascular injury (dissection, perforation, pseudoaneurysm formation, retroperitoneal hemorrhage, etc.), or distal embolization that may be induced by a delivery catheter, guidewire, or vascular sheath [50]. They are associated with worse outcomes at 30-day and one-year follow-up, including life-threatening bleeding and all-cause death. The rates of vascular complications have declined significantly from the time of initial commercial approval (15% to 22%) [65,66], mostly due to improving technologies with smaller delivery systems, optimization of procedural planning with preprocedural imaging, and increasing operator expertise [65]. Indeed, introduction of second- and third-generation systems that use smaller sheath sizes (18, 16, and 14 Fr) has significantly decreased the rates of vascular access-related complications. Moreover, low-profile expandable introducer sheaths have been recently released, which are designed to reduce the longitudinal forces on the artery. Therefore, further iterations in the design and size of the bioprosthetic valve and delivery catheter is expected to reduce sheath size and thus decrease the rate of vascular complications.

### 3.6. Conduction Disturbances

Conduction disturbances, mainly atrioventricular block requiring permanent pacemaker implantation (PPI) or new onset left bundle branch block, remain one of the most frequent device-related complications after TAVR and are associated with increased one-year mortality and HF- hospitalization [67]. Complete heart block requiring PPI within 30 days still occurs in around 10–15% of patients undergoing TAVR, even with newer generation devices. Despite notable advances in THV design and procedural technique, the rates of post-TAVR conduction disturbances have not significantly decreased over time, ranging from 4.0% to 24.0% with the Sapien 3 and from 14.7% to 26.7% with the Evolut R [68], even in the most recent years [69,70].

## 4. Clinical and Anatomic Factors to Consider in Bioprosthesis Selection

Table 1 displays the main features of the most common THV and Table 3 their potential advantages in specific scenarios, which are further examined in the subsequent sections.

### 4.1. Age and Life Expectancy

Currently, a number of randomized trials have clearly shown that TAVR is at least as safe and effective as SAVR for patients at low surgical risk [8,9,71]. This has led to the expansion of TAVR indications to younger patients, making it necessary to consider factors that may diminish long-term valve durability and life-expectancy. Similarly, the choice of THV implanted will unavoidably affect subsequent interventions in patients with greater life expectancy. Data on transcatheter bioprostheses durability are still scarce, despite data from prohibitive and high surgical risk cohorts indicating relative durability [72]. However, long-term head-to-head comparisons of the currently available devices are scarce, and several factors should be accounted for in the selection of the optimal device for patients with greater life expectancy. Future coronary access is a relevant aspect of lifelong care in this setting. Devices with lower stent frames and intra-annular leaflets have been shown to be less likely to cause coronary obstruction and may be preferred in younger patients. In addition, life expectancy might exceed valve durability in younger patients, and a strategy for possible TAVR-in-TAVR must be evaluated. Again, intra-annular bioprosthesis is less likely to impair coronary access in case of ViV.

Of note, the long-term consequences of conduction disturbances must be carefully considered in younger patients, as PPI are strongly associated with development of significant tricuspid valve regurgitation and right ventricular dysfunction, while bleeding, erosion, infection, and need for revision occur rarely. In general, the Evolut SE-THV have higher rates of post-TAVR conduction abnormalities as compared with BE-THV. The differences between the two valve types are related to the differing mechanisms of expansion as well as depth of implantation, which is deeper with the Evolut SE-THV and is known to be a strong predictor of post-TAVR PPI [73]. Anatomical and procedural features that predict the development of conduction disturbances and need for PPI include annular calcification, oversizing, left ventricular outflow tract eccentricity, and shorter septum length, but the strongest predictor is the presence of preprocedural conduction abnormalities, especially right bundle branch block [68]. Therefore, in patients presenting with these risk factors, especially in the case of long-life expectancy, bioprostheses with lower radial strength and limited extension to the membranous septum—such as the ACURATE neo and neo2—might be considered to minimize the risk of PPI. Nonetheless, recent studies failed to demonstrate the non-inferiority of the ACURATE THV in comparison with the CoreValve and Sapien THVs [74,75], and only limited evidence is currently available in support of the ACURATE neo2 THV [76]. Lastly, there is increasing evidence that intra-annular devices are more commonly affected by subclinical leaflet thrombosis, which in turn have been considered a strong risk factor for cerebrovascular accident and might increase the risk of structural valve deterioration. More data are needed to define the association between intra-annular design and subclinical leaflet thrombosis, as well as procedural and pharmacologic strategies to prevent subclinical leaflet thrombosis, and the clinical implications thereof.

### 4.2. Presence, Severity, and Disposition of Calcification

The advent of computed tomography scans helped in recognizing the importance of calcification severity and disposition in determining the risk for periprocedural adverse events, PVL, and valve deterioration. Based on calcification presence and disposition at the leaflets, annulus, LVOT, and sinus of Valsalva level, a wide range of potential drawbacks must be considered.

For instance, LVOT or annular asymmetric calcification may limit circular bioprosthesis expansion, often resulting in significant PVL, especially when a bioprosthesis without an outer sealing skirt is implanted. Similarly, severe or bulky isolated LVOT or annular calcification remarkably increases the risk of annular rupture, especially in case of BEV implantation. Lastly, the presence of severe and asymmetric LVOT calcification, especially when located below the left coronary cusp, due to the high probability of asymmetrical expansion of THV toward the commissure between right and noncoronary cusp, close to the His bundle [77]. Despite the lack of strong evidence supporting their use, mechanically expandable valves were considered a valid option in patients with a severe calcific annulus, especially if intolerant to rapid pacing or with a prior PPI. Of note, limiting the depth of implantation of SE-THV is another option that has been shown to reduce the risk of PPI [78].

### 4.3. Aortic Annulus, LVOT and Septum Characteristics

The optimal size of bioprostheses is largely dependent on aortic annular and root dimensions. Unfortunately, most devices are available with diameters ranging from 20 to 29 mm, but only with 3-mm increments. A notable exception is represented with the Myval BE-THV, available with diameters ranging from 20 to 32 mm and with 1.5-mm increments. The lack of intermediate diameters often results in valve oversizing, which is defined as a stent frame area 20% larger than annulus area and is another major predictor of annular rupture [79]. Hence, in the presence of other risk factors for annular rupture, the availability of a device with specific diameter might guide the selection of the bioprosthesis. Indeed, for annular dimensions that fall between two valve sizes, the choice is between a larger valve, which allows to optimize hemodynamic performance, and an undersized valve, in order to limit the risk for aortic root injury, as in case of significant annular and LVOT calcification. A small multicenter study including patients receiving a Sapien 3 Ultra BEV recently reported a comparison among extreme annular undersizing and nominal annular sizing. The authors reported no clinically meaningful difference at short-term follow-up in terms of valve hemodynamic performance. As there is a lack of recommendations guiding the choice between a smaller or larger equivalent valve in patients with borderline annular sizing, the authors speculate that extreme annular undersizing with the S3U transcatheter aortic valve might be a valuable option in this setting, as it limits the risk of annular rupture and its associated mortality risk, especially in the case of moderate or severe annular or LVOT calcification [80].

Conversely, more evidence is available with regard to patients with small aortic annulus. A small aortic annulus—usually defined as a perimeter < 72 mm, or an area < 400 mm^2^—is a well-defined risk factor for PPM [81], especially in case of concomitant large body surface area, while the rate of significant PVL seems to be low in patients with small aortic annulus. In these patients, or in those needing ViV procedures, there is consistent evidence from observational and randomized studies supporting the use of a supra-annular device, in order to ensure a larger EOA [82,83]. In addition, post-dilation and higher implantation depth should be considered in such cases. Conversely, balloon-expandable valves are particularly indicated in patients with large annulus diameter. In more detail, in patients with large aortic annuli, defined according to an area of 575–683 mm^2^ or a perimeter of 85.0–94.2 mm, the choice is limited to the Evolut R SE-THV, available with a diameter up to 34 mm, and the 29-mm Sapien 3, as its balloon may be overfilled with good results. Of note, the use of any valve is considered off-label in case of very large aortic annulus, and there is a paucity of data on the outcome of patients with very large aortic annulus treated with TAVR, mainly limited to observational studies. The TAVR-LARGE Registry reported promising results in this setting with both the 29-mm Sapien 3 and the 34-mm Evolut R THVs, although valve embolization, need for second valve implantation, and PVL occurred more frequently in patients receiving the 34-mm Evolut R, resulting in lower device success [84]. A retrospective analysis from the ACC/STS U.S. TVT registry LVOT reporting the outcome of 74 patients with very large aortic annulus treated with the 29-mm Sapien 3 BEV showed consistent findings, with low rate of adverse events and good hemodynamic performance. Of note, the presence of larger LVOT was associated with increased risk of PVL, especially in case of LVOT area larger than annular area, probably related to reduced LVOT sealing, suggesting that the 34-mm Evolut R might be considered in these cases.

Sigmoid septum bulging in the LVOT is another anatomical feature commonly seen in elderly and hypertensive patients with severe AS that might impair TAVR procedural success. Indeed, a sigmoid septum may interfere with stable catheter deployment and result in THV embolization, a complication commonly referred to as “watermelon seeding”. In addition, sigmoid septum is a well-defined risk factor for atrio-ventricular block and development of dynamic LVOT obstruction [85]. In this setting, some authors suggest preferring SEV with or without lower implantation height, in order to improve valve stability, although this benefit is counterbalanced by an increased likelihood of the need for PPI [86]. Another option, although only described by case reports and small case series and encumbered by similar risk of need for PPI [87,88], is represented by alcohol septal ablation and septal myectomy before proceeding with TAVR.

Lastly, membranous septum length should be carefully evaluated during preprocedural planning, as it might be considered as an anatomic surrogate of the distance between the aortic annulus and His bundle [89]. In more detail, the difference between membranous septum length and implantation depth has been consistently shown to be directly related to increased risk of need of PPI and of PM dependency at mid-term follow-up [77]. Hence, minimizing the overlap between membranous septum and the prosthesis frame, especially if a SE-THV with high radial force like the Evolut SE-THV has been selected, might help in reducing the risk of PPI [90].

### 4.4. Vascular Anatomy

Since the introduction of TAVR, there has been an impressive reduction in the dimensions of device insertion profiles. Early transcatheter bioprostheses required insertion sheath large up to 24 Fr, resulting in high risk for vascular complications and common use of alternative access to the femoral artery. At this time, an iliofemoral luminal diameter ≥ 5 mm is generally required for transfemoral TAVR, as the reduced profile of insertion sheath, coupled with the development of expandable sheaths such as the iSleeve sheath (Boston Scientific, Marlborough, Massachusetts, USA), allowed for transfemoral approach in a larger proportion of patients and limits the risk of vascular complications. Nonetheless, the prevalence of severe iliofemoral or aortic disease among patients undergoing TAVR is considerable, and significant tortuosity, calcification, atheroma, and aortic thrombus apposition must be evaluated. In these cases, a delivery system that allows for flexion of the distal catheter system would be preferable, in order to limit aortic wall trauma or embolization at level of the aortic arch and to attenuate the risk of periprocedural stroke. A flexible delivery system may also be indicated in the case of a horizontal aorta, defined as an aortic angulation >60°. An example of this is the Edwards Commander delivery system, available for use with the Sapien 3 BE-THV.

## 5. Knowledge Gaps

In the last several years, head-to-head comparisons between different devices have helped identifying the scenario(s) in which each device could perform optimally. Nonetheless, these studies were commonly too underpowered to allow for meaningful subgroup analyses, as well as the relatively low rate of adverse events, which impelled the need for complex composite end points to detect significant differences in clinical outcomes among groups. Hence, future studies comparing different devices could be focused on specific outcomes and subgroup of patients, in order to shed light on the performance of each device in patients with clinical and anatomical peculiarities and to help in moving from a “one valve fits all” to a patient-tailored selection of the device.

In addition, long-term outcomes, such as valve durability, are complicated to assess, considering the long-term follow-up required and the continuous development of new devices iterations, which would have replaced previous devices by the end of study follow-up.

## 6. Conclusions

The continuous evolution in TAVR devices and materials, coupled with improvements in operators’ skills and procedural techniques, allowed the expansion of TAVR indications to patients across the spectrum of surgical risk. This led to broadened heterogeneity in patient clinical and anatomical characteristics that mandate careful consideration when selecting the optimal device for each patient, in order to continuously improve predictability, safety, and efficacy of TAVR.

## Figures and Tables

**Table 1:** Overview of Transcatheter Aortic Valve Replacement (TAVR) Prostheses.

Prosthesis	Frame Material	Leaflet Material	Valve Sizes (mm)	Sheath Sizes	Supra- or Intra- Annular	Repositionable/Retrievable	Delivery Routes	FDA Approval	CE Mark Approval
Balloon-expandable									
Sapien	Stainless steel	Bovine pericardium	23, 26	22F (23 mm), 24F (26 mm)	Intra-annular	No/No	TF, TA	✓	✓
Sapien XT	Cobalt-chromium	Bovine pericardium	23, 26, 29	16F (23 mm), 18F (26 mm), 20F (29 mm)	Intra-annular	No/No	TF, TA, TAo	✓	✓
Sapien 3	Cobalt-chromium	Bovine pericardium	20, 23, 26, 29	14F (20, 23, 26 mm), 16F (29 mm)	Intra-annular	No/No	TF, TA, TAo	✓	✓
Sapien 3 Ultra	Cobalt-chromium	Bovine pericardium	20, 23, 26, 29	14F	Intra-annular	No/No	TF	✓	✓
Myval THV	Nickel-cobalt	Bovine pericardium	20, 23, 26, 29, 21.5, 24.5, 27.5, 30.5, 32	14F	Intra-annular	No/No	TF		✓
Self-expanding									
CoreValve	Nitinol	Porcine pericardium	23, 26, 29, 31	18F	Supra-annular	Yes/Yes	TF, TAo, SC	✓	✓
Evolut R	Nitinol	Porcine pericardium	23, 26, 29, 34	14F (23, 26, 29 mm), 16F (34 mm)	Supra-annular	Yes/Yes	TF, TAo, SC	✓	✓
Evolut PRO	Nitinol	Porcine pericardium	23, 26, 29, 34	16F	Supra-annular	Yes/Yes	TF, TAo, SC	✓	✓
Evolut PRO+	Nitinol	Porcine pericardium	23, 26, 29, 34	14F (23, 26, 29 mm), 16F (34 mm)	Supra-annular	Yes/Yes	TF, TAo, SC	✓	✓
ACURATE neo	Nitinol	Porcine pericardium	23, 25, 27	18F	Supra-annular	No/No	TF, TA		✓
ACURATE neo2	Nitinol	Porcine pericardium	23, 25, 27	14F	Supra-annular	No/No	TF, TA		✓
Allegra	Nitinol	Bovine pericardium	23, 27, 31	18F	Supra-annular	Yes/Yes	TF		
Hydra	Nitinol	Bovine pericardium	22, 26, 30	18F	Supra-annular	Yes/Yes	TF		✓
Engager	Nitinol	Bovine pericardium	23, 26	30F	Supra-annular	Yes/Yes	TA		✓
Venus-A valve	Nitinol	Porcine pericardium	23, 26, 29, 32		Supra-annular	Yes/No	TF		
VitaFlow	Nitinol	Bovine pericardium	21, 24, 27, 30	16F (21, 24 mm), 18F (27, 30 mm)	Supra-annular	Yes/No	TF, TAo, CA		
VitaFlow Liberty	Nitinol	Bovine pericardium	21, 24, 27, 30	16F (21, 24 mm), 18F (27, 30 mm)	Supra-annular	Yes/No	TF, TAo, CA		
Centera	Nitinol	Bovine pericardium	23, 26 29	14F	Intra-annular	Yes/Yes	TF		✓
Portico	Nitinol	Bovine pericardium	23, 25, 27, 29	18F (23, 25 mm), 19F (27, 29 mm)	Intra-annular	Yes/Yes	TF, TAo, TAx, SC		✓
Navitor	Nitinol	Bovine pericardium	23, 25, 27, 29	14F (23, 25 mm), 15F (27, 29 mm)	Intra-annular	Yes/Yes	TF, TAo, TAx		✓
Mechanically expandable									
Lotus	Nitinol	Bovine pericardium	23, 25, 27	20F (23, 25 mm), 22F (27 mm)	Intra-annular	Yes/Yes	TF, TAo	✓	✓
Lotus Edge	Nitinol	Bovine pericardium	23, 25, 27	15F	Intra-annular	Yes/Yes	TF, TAo	✓	✓
Lotus Mantra	Nitinol	Bovine pericardium	23, 25, 27	12F	Intra-annular	Yes/Yes	TF, TAo	✓	✓
Aortic regurgitation									
JenaValve	Nitinol	Porcine pericardium	23, 25, 27	19F	Intra-annular	Yes/Yes	TA		✓
J·Valve	Nitinol	Bovine pericardium	22, 25, 28	18F	Intra-annular	No/No	TA		✓

TF-Transfemoral, TA-Transapical, TAo-Transaortic, TAx-Transaxillary, SC-Subclavian, CA-Carotid. FDA Approval–approved for use by the United States Food and Drug Administration. CE Mark Approval–approved for use across all EU member states, European Econamic Area, and Turkey by the European Commission. ✓ = approved.

**Table 2:** Rate of adverse events after transcatheter aortic valve replacement (TAVR) and surgical aortic valve replacement (SAVR) reported in randomized controlled trials.

Event	TAVR	SAVR	Follow-Up
Stroke	0.6–6.7%	2.4–6.1%	30 Days
	4.1–10.6%	4.3–8.7%	1 Year
Subclinical Leaflet Thrombosis	13%	5%	30 Days
	28%	20%	1 Year
Coronary Obstruction	0.2–1.7%	0–0.6%	
Severe Prosthesis-Patient Mismatch	9.3–12%	27.8%	
Clinically Significant Paravalvular Leak	0.5–13.6%	–	
Vascular Complications	3.8–30.7%	1.1–11.3%	30 Days
Conduction Disturbances (PPI)	3.4–34.1%	1.6–7.1%	30 Days

PPI–Permanent pacemaker implantation.

**Table 3:** Selection of optimal bioprosthesis in different clinical scenarios.

	Balloon-Expandable	Self-Expanding
Clinical factors		
Greater life expectancy	✓	
Heart failure		✓
Chronic kidney disease		✓
Pre-existing or risk ^#^ for conduction disturbances	✓	
Anatomic features		
Small annulus	✓	✓ (supra-annular)
Large annulus	✓	
Dense annular calcification		✓
Need for coronary access	✓	
Horizontal aorta *	✓	
Valve-in-Valve		✓

* aortic angulation >60°. ^#^ left ventricular outflow tract eccentricity, shorter septum length. ✓ device recommended.

## Data Availability

Not applicable.
